# Having chronic back pain did not impact COVID-19 outcome in a low-income population – a retrospective observational study

**DOI:** 10.1186/s42358-023-00347-7

**Published:** 2024-01-11

**Authors:** Antônia Celia de Castro Alcantara, Hermano Alexandre Lima Rocha, Jobson Lopes de Oliveira, Xenofon Baraliakos, Francisco Airton Castro Rocha

**Affiliations:** 1 Postgraduate Program in Medical Sciences, Federal University of Ceará, Fortaleza, Brazil; 2 Department of Maternal and Child Health, Federal University of Ceará, Fortaleza, Brazil; 3 Rheumazentrum Ruhrgebiet Herne, Ruhr-University Bochum, Bochum, Germany; 4 Instituto de Biomedicina – Laboratório de Investigação em Osteoartropatias, Rua Coronel Nunes de Melo, 1315 -1º. Andar Rodolfo Teofilo, Fortaleza, CE, CEP: 60430-270 Brazil

**Keywords:** Back pain, COVID-19, Epidemiology, Spondyloarthritis

## Abstract

**Introduction:**

Chronic back pain (CBP) is a major cause of years lived with disability. Social inequalities increase the prevalence and burden of CBP. Management of CBP was affected by restricted access to non-pharmacological treatments and outdoor activities during COVID-19 pandemic.

**Objective:**

To determine the prevalence of CBP among patients with COVID-19 as well as the impact of having CBP in COVID-19 outcome in our low-income population.

**Methods:**

Retrospective cohort of individuals with confirmed COVID diagnosis from May 2020 - March 2021, at Hospital Regional UNIMED (HRU) in Fortaleza, Ceará, Brazil. Data included comorbidities and household income.

**Results:**

Among 1,487 patients, 600 (40.3%) were classified as having CBP. Mean age as well as income were similar in CBP and non-CBP groups, with more women in the CBP group. Hypertension and asthma, but not diabetes, were more prevalent in those with CBP. Need for emergency care, hospitalization, and admission to intensive care unit were similar regardless of having CBP. Dyspnea was more common in CBP vs. non-CBP groups, with 48.8% vs. 39.4% percentages, respectively (*p* = 0.0004).

**Conclusion:**

Having CBP prior to COVID did not impact the acute clinical outcome of COVID individuals of a low-income population.

**Supplementary Information:**

The online version contains supplementary material available at 10.1186/s42358-023-00347-7.

## Introduction

The impact of COVID-19 pandemic to health care systems globally is still underestimated. In addition to the catastrophic number of deaths linked to this viral infection, long term sequelae associated with this disease have severely impacted many individuals that survived the pandemic [1, 2]. The extended lockdown implemented in most countries brought changes in lifestyle and daily life activities that severely affected both prevention and treatment of other disease states. A major consequence of the lockdown policy was the limitation of physical and social activities, thus increasing sedentary behavior as well as worsening mood disorders [3–5]. The pathophysiology of chronic back pain (CBP) has been linked to a multitude of psychological, physical, social, and economical factors, which were collectively encompassed by a “bio-psycho-social-model” [6]. Treatment approach for CBP relies mostly in the practice of regular physical activity, management of underlying mood disorders, and measures to prevent damage to the back related to professional issues. Such measures were compromised due to COVID-19 lockdowns. In our region, access to outpatient services, including recreational and fitness facilities, was severely restricted. Furthermore, patients were strongly advised to stay at home and not to seek medical care for COVID-19 unless feeling severely ill [5, 7–9]. A recent systematic review with meta-analysis found that low back pain prevalence and intensity significantly increased during the COVID-19 lockdown, as compared to the pre-pandemic period, with women being more frequently involved [10]. Risk factors associated with low back pain include smoking habit, diabetes and physical inactivity, which are also linked to more severe COVID-19 [11]. We have recently reported the prevalence of CBP as well as inflammatory back pain in our low-income population [12]. Our aim was to determine the prevalence of CBP in patients with COVID-19 and assess whether presence of CBP could impact short term COVID-19 outcome in our population.

## Methods

### Study design

This was a retrospective observational cohort study conducted from May 2020 to March 2021, at the Hospital Regional UNIMED (HRU) in Fortaleza, Ceará, Brazil. In addition to the hospital’s emergency room (ER), a virtual emergency room (VER) was implemented to care for patients with suspected symptoms of COVID-19 on February 17, 2021, initially in a remote mode, aiming to decrease the need for visits to the ER for those with milder symptoms while at the same time providing patient support in a timely manner. The decision to seek remote assistance was at the discretion of the patient or caregiver, using a specific mobile device, named “Cliente Unimed Fortaleza”, providing free daily access to board certified physicians specifically trained for this project, as per regulations of the Associação Médica Brasileira [9].

### Patient selection

Eligible patients were selected among individuals presenting to the ER or VER of the HRU. Inclusion criteria were age over 18 with clinical suspicion of COVID-19, followed by confirmation with a positive polymerase-chain reaction for SARS-Cov-2 in nasal swab samples. Data generated from March 2020 to March 2021 were collected directly from the medical records to include patients reporting CBP, meaning pain persisting over 3 months duration, gathering data on demographics, socioeconomic, and clinical information of all patients admitted either to this outpatient remote care or to the emergency facilities of the HRU. Individuals with medical records either lacking confirmation of COVID-19 diagnosis or information on presence/absence of CBP were excluded.

### Comorbidities, symptoms, and socioeconomic data

Information on comorbidities included self-reported history of systemic arterial hypertension, cardiovascular disease, diabetes, and asthma. Patients were specifically questioned regarding presence of smell/taste disorders, cough, and odynophagia starting after the appearance of the first signs of COVID infection. Fever considered occurrence of measured axillary temperature over 37.5 °C. Presence of dyspnea was registered by physicians during the first visit to the ER or using the implemented VER. Socioeconomic data included self-reported monthly household income using March 2020 as a reference for converting Brazilian currency R$ to US$, using the monthly minimum wage, as follows: </≥3 minimum wages, which corresponded approximately to below 730.00 US$ and above 730.00 US$.

### Statistics

Data are presented as means or percentages, as appropriate, evaluating individuals with a confirmed COVID-19 diagnosis. Normality was assessed using Kolmogorov-Smirnov test. Categorical variables were compared using chi-Square. Values of *p* < 0.05 were considered significant. Analysis performed using IBM software, SPSS Statistics for Windows, Version 23.0. Armonk, NY: IBM Corp. IBM Corp. Released 2015. This protocol was approved by the Unichristus Ethics Committee that follow the guidelines of CONEP, the Brazilian system for ethical assessment of human research.

## Results

### Demographics and socioeconomic data

There were 600 (40.3%) individuals with CBP among 1,487 patients with a COVID diagnosis. Age was similar regardless of having CBP with more women having CBP, as shown in Table 1. Family income was slightly though not significantly higher in those without CBP. Hypertension and asthma, but not diabetes, were significantly more prevalent in patients with CBP (Table 1).

**Table 1:** Gender, income, and prevalence of comorbidities in the presence of chronic back pain among COVID-19 affected patients in a low-income population

		CBP (600)	Non-CBP (887)	*p-value*
Gender	Male	167 (27)	385 (43)	< 0.001
	Female	433 (73)	502 (57)	
Age	Mean	44.91	44.04	
Family income	≥ 730 US$	338 (56)	488 (58)	0.06
	< 730 US$	291 (44)	343 (42)	
Hypertension	Yes	172 (28)	186 (20)	< 0.0006
	No	417 (72)	687 (80)	
Asthma	Yes	59 (9)	61 (6)	0.041
	No	528 (91)	805 (94)	
Diabetes	Yes	75 (12)	109 (12)	0.889
	No	510 (88)	758 (88)	

Table 1. Demographic, income, and comorbidity data were collected from 1487 COVID-19 affected patients living in a low-income region. Data represent N(%) compared using bivariate analysis (missing information in all but gender data)

### COVID outcome

Clinical outcome of COVID patients of the CBP and non-CBP groups is shown in Table 2. There was a mild though statistically significant higher number of non-CBP individuals seeking emergency care, as compared to those with CBP. On the other hand, the percentage of individuals with CBP that needed hospitalization because of worsening COVID-19 was numerically higher as compared to that of non-CBP individuals. This was also mirrored by a trend towards more patients of the CBP group needing admission to intensive care unit, though not reaching statistical significance (Table 2).

**Table 2:** Demand for emergency service, hospitalization, and admission to intensive care unit among COVID-19 with and without CBP

		CBP (600)	Non-CBP (887)	*P-value*
Emergency Care	No	547 (91.17)	809 (90.3)	0.030
	Yes	53 (8.83)	78 (9.64)	
Hospitalization	No	543 (92.2)	812 (93.0)	0.162
	Yes	45 (7.7)	62 (6.9)	
Intensive Care	No	555 (83.3)	825 (93.0)	0.0800
	Yes	4 (0.66)	5 (0.56)	

Data represent N(%), comparing CBP and non-CBP groups using chi-Square; CBP, chronic back pain.

### Symptoms related to COVID-19

Alterations of smell/taste and headache were the most common symptoms reported by patients with COVID-19 diagnosis. All symptoms commonly attributed to COVID-19, but dyspnea, were similarly present regardless of a CBP diagnosis. There was also a trend for a greater prevalence of headache in the CBP group (Table 3).

**Table 3:** Association of COVID-19 symptoms with presence of CBP

		CBP (600)	Non-CBP (887)	*P-value*
Dyspnea	Yes	282 (48.8)	337 (39.4)	0.0004
	No	296 (51.2)	519 (60.6)	
Smell/Taste disorders	Yes	452 (77.0)	673 (78.2)	0.6017
	No	135 (23.0)	188 (21.8)	
Headache	Yes	434 (73.7)	601 (69.0)	0.0533
	No	155 (26.3)	270 (31.0)	
Fever	Yes	368 (62.5)	557 (63.9)	0.5672
	No	221 (37.5)	314 (36.1)	
Cough	Yes	357 (60.5)	500 (57.1)	0.5672
	No	233 (39.5)	376 (42.9)	
Odynophagia	Yes	263 (45.5)	365 (42.4)	0.2514
	No	315 (54.5)	495 (57.6)	

Prevalence of the most common COVID-19 symptoms in patients with/without CBP. Data represent N (%), comparing CBP vs. non-CBP using chi-Square; CBP, chronic back pain.

## Discussion

Our data showed a prevalence of 40.3% of CBP among patients with a COVID-19 diagnosis, which is similar to the 43% prevalence of CBP we have recently reported among individuals in the same region prior to the pandemic [12]. Having a CBP diagnosis apparently did not impact COVID-19 outcome in our low-income population. Although there are studies reporting the prevalence of musculoskeletal symptoms in patients affected by COVID-19 and others addressing low back pain in patients with COVID-19 [10, 11], we are not aware of previous data reporting the prevalence of CBP among COVID-19 patients. Neither had the impact of having CBP in COVID outcome been described. Our COVID patients with CBP were more commonly affected by hypertension and asthma. The increased prevalence of these comorbidities might have been influenced by the fact that such patients, when affected by COVID-19, usually carry a higher chance of developing worse disease [9]. In this case, given that we selected patients visiting an emergency room, it is likely that hypertensive and asthmatic individuals with COVID-19 would seek earlier health care whereas previously healthy individuals might have more commonly being asymptomatic or present a milder disease. Additionally, access to remote orientation probably encouraged those patients with comorbidities to look for medical care [9, 13–16].

The frequency of COVID-19 symptoms reported in our cohort was similar to that of previous studies with smell/taste disorders, headache, fever, odynophagia, and cough being the most frequent [16–19]. All common early COVID-19 symptoms but dyspnea were similarly present in our cohort, regardless of being classified as having CBP. There have been studies reporting a high prevalence of musculoskeletal symptoms in patients affected by COVID-19, as well as in the post-COVID-19 syndrome [20]. Comparing to wealthier regions, we can consider our entire cohort to be of low-income, given that a large percentage of individuals in both groups declared monthly household incomes below $730.00. Prevalence of CBP has been associated with lower income, especially among individuals with blue-collar jobs [21, 22]. However, our numbers reporting CBP prevalence, regardless of having COVID-19, seem similar to data from developed regions [12]. This may question the relevance of mean income per se, at least in urban regions, as a factor influencing presence of CBP. However, this remains to be demonstrated. Cardiovascular and metabolic comorbidities have been shown to lead to a worse COVID-19 outcome [15, 16]. In our study, hypertension and asthma were more frequent among patients with COVID-19 reporting CBP. Interestingly, although there were more non-CBP individuals seeking the emergency care, CBP individuals had a trend towards requiring more hospitalization and admission to an intensive care unit, though not reaching statistical significance. Although we believe that the low number of individuals in those groups probably have impacted statistical analysis, our data indicate that having CBP did not affect COVID-19 outcome. Common symptoms related to COVID-19 infection, but dyspnea, were equally frequent among our patients, regardless of the presence of CBP. Unfortunately, we cannot discuss whether having dyspnea and CBP affected COVID-19 outcome, since we were not able to track down the patients according to the presence of dyspnea at admission. However, we may speculate that such individuals might have had more severe COVID-19, thus possibly accounting for a higher number of CBP individuals being hospitalized and admitted to intensive care. Although being the first report on the possible impact of CBP in COVID-19 outcome, particularly in a low-income cohort, our study has some limitations. One is represented by the fact that we did not capture data from all COVID-19 patients. The vast majority of patients probably had milder disease thus not seeking medical advice. Fear for coming to an emergency room was common during COVID-19 [9]. While this could have been facilitated by the remote access, we believe most individuals with mild symptoms might not have felt necessary to ask for medical help. It would also be interesting to document whether having had COVID-19 affects the prevalence of CBP after recovery from the infection. We also did not collect data on the prevalence of inflammatory back pain among CBP individuals, as we done in our previous cohort [12, 23]. This was caused by the fact that many individuals with COVID were already taking nonsteroidal anti-inflammatory drugs as well as oral corticosteroids, which can be obtained without a medical prescription in our region.

In conclusion, we present the first data regarding prevalence and clinical impact of having CBP in individuals with COVID-19 infection. Comorbidities associated with a worse prognosis in this viral disease, namely hypertension and asthma, were also more common among individuals with CBP [16]. Though dyspnea, which has been associated with more severe COVID-19 [1], was also more common in those patients with CBP, our data indicate that having CBP did not have an impact in COVID-19 outcome in our low-income population.

## Electronic supplementary material

Below is the link to the electronic supplementary material.

Supplementary Material 1

Supplementary Material 2

## Data Availability

All data generated or analysed during this study are included in this published article.

## Abbreviations

CBP: Chronic back pain
ER: Emergency room
HRU: Hospital Regional Unimed
OA: Osteoarthritis
VER: Virtual Emergency Room
