# Supplementary material for: Having chronic back pain did not impact COVID-19 outcome in a low-income population – a retrospective observational study
Source: Adv Rheumatol. 2024 Jan 11;64:7. doi: 10.1186/s42358-023-00347-7 (PMC13488761; doi:10.1186/s42358-023-00347-7)
Supplement: Supplementary file 1 [file 42358_2023_347_MOESM1_ESM.docx]

**Answers to Reviewers**

**Comment:**

Reviewer #1: Dear researchers,

Thank you for the opportunity to review your manuscript entitled "Chronic back pain did not impact COVID-19 outcome in a low-income population."
The manuscript is good, but the authors would need to address some significant issues before they can publish it.

Some sections are difficult to follow. Thus you need to reordered/rewritten following  the guidelines for reporting observational studies "The Strengthening the Reporting of Observational Studies in Epidemiology (STROBE) Statement". Therefore, you should add all information, following the STROBE checklist, in each section of this manuscript.

**Answer:** We are very grateful for the careful revision made, which will improve the quality of our study. The text was thoroughly revised and structured using the STROBE recommendations, starting with the title and abstract. Please note that every added part of the text in the marked copy appears in red, while deleted parts appear **bold.** Particularly, please see changes in the methods and results section.

**Comment:**
Introduction
Lines 66-72: It is not very clear why we discuss the treatment of osteoarthritis. You should only describe the usual treatment prescribed for CLBP. After explaining the most common treatment choices to treat CLBP, you should focus your attention on patients and why they cannot access usual care services due to Covid-19. This could make the Introduction more linear.

**Answer:** the sentence making reference to OA was omitted. We added a mention to some lockdown measures implemented in our place, which severely restricted access to non-pharmacological health care, as follows: Parte superior do formulário

In our region, significant limitations were imposed on all outpatient services, in addition to restriction of access to recreational and fitness facilities. Also, individuals were advised to stay at home and refrain from going to emergency rooms unless feeling severely ill. Please see lines 66-68, page 3 of the revised version.

**Comment:**
Methods
Please explain in detail what COVID-19 symptoms are analyzed, shown in Table 2.
Provide eligibility criteria, as well as participant selection sources and methods.
**Answer:** Detailed information regarding collection of data regarding symptoms now appears in the methods sections, as follows: *Patients were questioned regarding presence of recent smell/taste disorders, cough, and odynophagia starting after the appearance of the first signs of COVID infection. Fever was registered considering axillary temperature over 37.5°C. Presence of dyspnea was registered by physicians during the first visit to the ER or VER.* Please see lines 102-106, page 5 of the revised version.

Inclusion and exclusion criteria are now emphasized, as follows: *Eligible patients were selected among individuals presenting to the ER or VER of the HRU. Inclusion criteria were age over 18 with clinical suspicion of COVID-19, followed by confirmation with a positive polymerase-chain reaction for SARS-Cov-2 in nasal swab samples.* Please see lines 90-99, page 4 of the revised version.

**Comment:**
Results
The results in this section are not well and clearly described. Divide this paragraph into subsections. Please report all results in tables 1-2-3 with number, percentages, and p-values.

**Answer:** this section was divided in 3 subsections, as requested. Missing information regarding percentage on income data in table 2 were entered.

**Comment:**
Discussion
This section is well described.

Reviewer #2:

**Comment:** Thank you for your submission - the article is certainly relevant to the field, as it provides original data on a very prevalent condition and its behavior during the COVID-19 pandemic. Title is concise and representative;  materials and methods were completely described, including ethical considerations, and data were clearly presented, addressing the objective proposed and also stating the limitations of the study. Therefore, I have few observations/doubts to be cleared. Please, answer them, as needed:
**Answer:** We are very grateful for the valuable comments and the time reviewing our study. We tried to address each and every question, hoping to meet all requests.

**Comment:**
1) Correct English grammar/spelling in line 175: While this could have been facilitated by the remote access, we believe most individuals with mild symptoms might not have FELT necessary to ask for medical help.

2) Add 'virtual emergency room (VER)' to the list of abbreviations.
**Answers:** The correction was made. VER was added to the list of abbreviations.

**Comment:**
3) The authors mentioned in the Introduction the ReumaCOV study, reporting no influence of COVID-19 in spondyloarthritis patients and vice-versa. How does that information correlate to the paper submitted? Considering the 600 individuals with CBP and COVID-19 studied, how many had the diagnose of spondyloarthritis? Could this subgroup of patients had avoided ER for fear, but when infected by coronavirus present more symptoms and need for hospitalization due to immunosuppression? Could these data be analyzed? If not, there is no reason to address spondyloarthritis in the introduction.

**Answer:** Many patients were using NSAIDs because of COVID symptoms, rendering it impossible to determine whether inflammatory back pain was present, which would be relevant for a spondyloarthritis diagnosis. Unfortunately, we did not specifically asked patients about this diagnosis. We omitted the mention to spondyloarthritis in the revised version. Please see that this part now appears **bold** in page 4 of the marked copy.

**Comment:**
4) The impact of COVID-19 pandemic in daily lives activities, such as physical activity, was brought to light. But no data nor discussion was made about  how many of the individuals in the sample had their non-pharmacological treatment affected and/or their condition worsened due to the pandemic. As this was not the objective of the paper, I strongly suggest to review and restructure the Introduction section so that it provides background on the purpose of the research.

**Answer:** The introduction section was reviewed trying to address this issue. Unfortunately, we could not control for this information, but most people stayed at home. The text was thoroughly reviewed and a mention to lockdown measures was included, as well as a sentence stating our aim, as follows: *Our aim was to determine the prevalence of CBP in patients with COVID-19 and assess whether presence of CBP could impact short term COVID-19 outcome in our population.* Please see lines 66-68 and 75-76, page 3 of the revised version.
