# Supplementary material for: Having chronic back pain did not impact COVID-19 outcome in a low-income population – a retrospective observational study
Source: Adv Rheumatol. 2024 Jan 11;64:7. doi: 10.1186/s42358-023-00347-7 (PMC13488761; doi:10.1186/s42358-023-00347-7)
Supplement: Supplementary file 2 [file 42358_2023_347_MOESM2_ESM.doc]

Fortaleza, November 18, 2023

**To the Editor:**

**Advances in Rheumatology**

#### Ref. Submission of a Revised version of Manuscript #ADRH-D-23-00150

Dear Editor,

We are submitting the electronic version of the manuscript entitled: **Having chronic back pain did not impact COVID-19 outcome in a low-income population – a retrospective observational study,** from **Alcântara et al.**, for publication in Advances in Rheumatology.

We are grateful for the comprehensive review of our article and the suggestions that surely help improve our manuscript. We answered each and every question raised, indicating in the uploaded marked copy where the modifications were made as well as in the revised file the lines and pages, in order to facilitate tracking the changes made. We hope we met all requests for approval of our manuscript.

On behalf of all authors, I hereby state that the material that is being submitted is not under consideration for publication elsewhere and that we have no conflicts of interest to disclose.

Yours sincerely,

Francisco Airton Castro da Rocha, MD, PhD.

Author for Correspondence

**Correspondence should be sent to:**

**Dr. Francisco Airton Rocha**

**Rua Cel. Nunes de Melo, 1315 – Rodolfo Teófilo**

**CEP 60430-270 - Fortaleza - CE – BRAZIL**

**Phone: ( 55 85) 33668243; FAX (55 85) 32446215; E-mail: arocha@ufc.br**
